# A Case of a Carotid Cavernous Fistula

**DOI:** 10.5811/cpcem.2022.1.55033

**Published:** 2022-04-25

**Authors:** Rami H. Mahmoud, Brooke A. Hensley

**Affiliations:** * University of Miami Miller School of Medicine, Miami, Florida; † University of Miami Miller School of Medicine, Jackson Memorial Hospital, Department of Emergency Medicine, Miami, Florida

**Keywords:** carotid cavernous fistula, Indirect carotid cavernous fistula, cavernous sinus, proptosis, transvenous embolization

## Abstract

**Case Presentation:**

A 73-year-old male presented to the emergency department complaining of pain in his right eye for four weeks. He denied any trauma, and the pain was accompanied by ptosis, proptosis, swelling, redness, blurred vision, and a frontal headache. On examination, conjunctival arterialization was also appreciated. Magnetic resonance imaging and angiography showed evidence of a carotid cavernous fistula for which the patient underwent successful transvenous coiling and embolization.

**Discussion:**

Carotid cavernous fistulas are classified as higher flow, direct fistulas or lower flow, indirect fistulas; the latter is more insidious in onset. Classic symptoms include conjunctival arterialization, proptosis, ptosis, palpebral edema, ocular palsy, vibratory sensation, elevated intraocular pressure without pupillary or visual acuity deficits, and headache. The treatment of choice is transvenous embolization.

## CASE PRESENTATION

A 73-year-old male with a past medical history of gastroesophageal reflux disease, hypertension, chronic obstructive pulmonary disease, and type 2 diabetes presented to the emergency department (ED) of our university hospital with a chief complaint of pain in his right eye for four weeks. This was accompanied by palpebral swelling, redness, blurred vision, and a frontal headache. The patient denied diplopia on presentation but slowly developed it over the course of his hospitalization. He also denied any trauma. He described the pain as constant, pulsatile and throbbing, and localized behind his right forehead; he denied prior episodes. The review of systems was otherwise negative, and social and family histories were noncontributory. The patient was taking 81 milligrams aspirin daily.

**Image 1:**
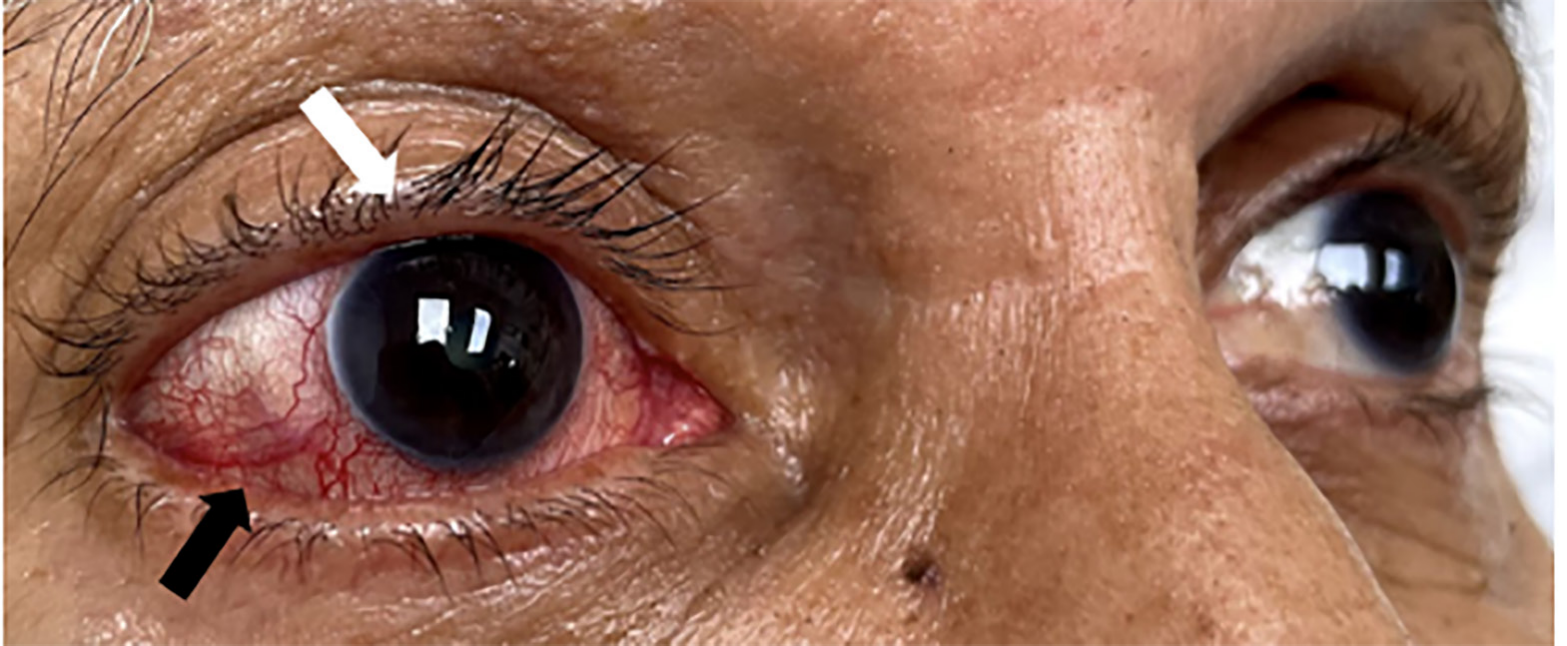
Conjunctival arterialization (black arrow) and ptosis as well as palpebral edema (white arrow) of the right eye.

**Image 2:**
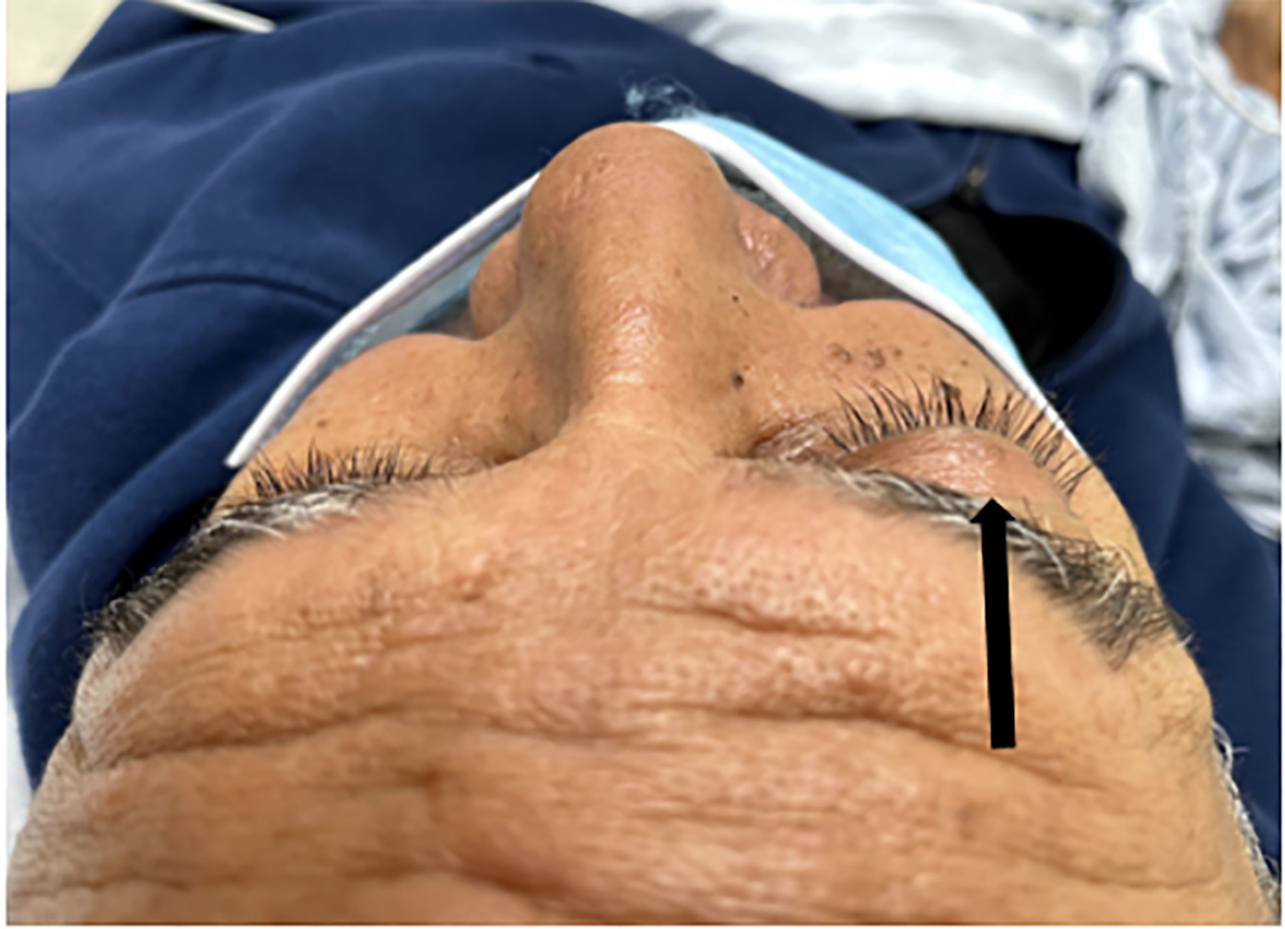
Proptosis of the right eye as visualized from above (arrow) in a case of carotid-cavernous fistula.

Physical examination revealed 20/20 and 20/25 visual acuity in the right and left eye, respectively. Intraocular pressure (reference range: 10–21 millimeters of mercury [mm Hg]) was 19 mm Hg in the right eye and 17 mm Hg in the left. The pupils were 3 mm and reactive to light bilaterally. Examination of the right eye was notable for scleral injection, proptosis, ptosis, and conjunctival arterialization or hyperemia (Images 1 and 2). Proptosis of the right eye is better visualized from above (Image 2). Extraocular movements were intact, and visual fields were normal. There were no additional cranial nerve deficits, ocular palsies, or facial numbness; and the patient was otherwise neurologically intact throughout. A computed tomography without contrast of the brain revealed mild involutional changes and mild periventricular hypodensities. Asymmetric enlargement of the right superior ophthalmic vein, fat stranding of the right orbit, proptosis of the right globe, and prominence of the right extraocular muscles were also appreciated. Magnetic resonance imaging (MRI) and angiography revealed the right middle meningeal artery merging into the right cavernous sinus, consistent with a cavernous carotid fistula (CCF).

## DISCUSSION

A CCF is an abnormal arterial venous connection within the cavernous sinus. Located lateral to the sella turcica, the cavernous sinus is a trabeculated venous cavity housed by the dura matter.^1^ Coursing through the cavernous sinus are several major neural and vascular structures, including the internal carotid artery (ICA) and cranial nerves III, IV, V1, V2, and VI.^1^ Carotid cavernous fistulas are classified as direct, characterized by a direct connection of the ICA and the cavernous sinus. or indirect, lower flow fistulas, where communications between cavernous arterial branches and the cavernous sinus are established.^1^,^2^

The Barrow classification, divided into four types (A through D), is used to specify the different anatomical variations of CCFs. Type A represents direct fistulas, and types B through D represent indirect fistulas.^1^,^2^,^3^,^4^ Type B is defined by connections between the dural branches of the ICA and the cavernous sinus, whereas type C fistulas are supplied by the dural branches of the external carotid artery (ECA).^1^,^2^,^3^,^4^ Angiography of the right and left ICAs in our patient revealed a Type D CCF, defined by involvement from the meningeal branches of the ICA and ECA.^1^,^2^,^3^,^4^

**CPC-EM Capsule**

What do we already know about this clinical entity?

*Carotid cavernous fistulas (CCF) may occur spontaneously or secondary to trauma. The treatment of choice is transvenous coiling and embolization*.

What is the major impact of the image(s)?

*These images bring attention to the presentation of CCFs, outlining characteristic clinical clues for timely diagnosis*.

How might this improve emergency medicine practice?

*Left unmanaged, CCFs may result in irreversible injury to the involved eye, highlighting the importance of a broad differential when presented with a common complaint (eye pain)*.

**Image 3:**
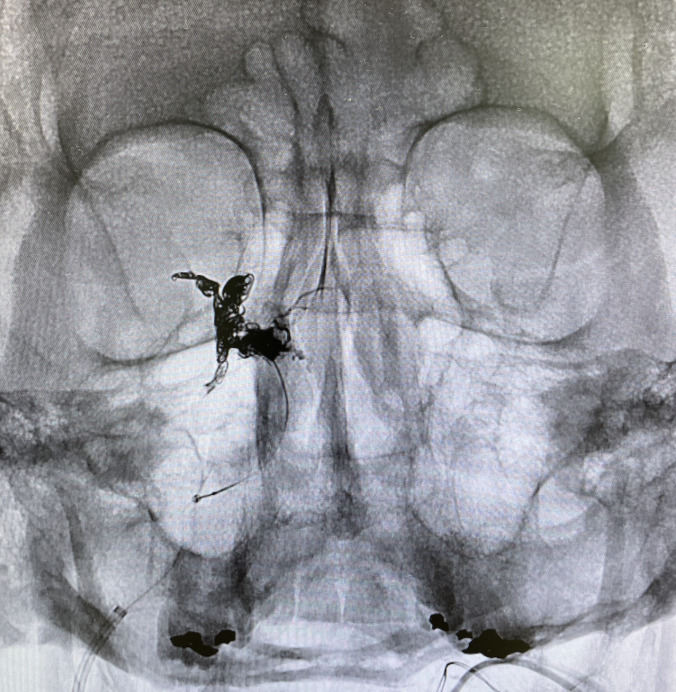
Post-operative imaging revealing successful coiling and embolization of the CCF.

Further classification of type D CCFs has been established with type D1 and D2 representing unilateral or bilateral arterial supply, respectively.^3^ Possible etiologies of indirect CCFs include hypertension, connective tissue disorders, and ICA dissections.^2^ Conjunctival arterialization is classically present (Image 1), with other common findings that may include chemosis, proptosis, diplopia, and ophthalmoparesis.^1^ The gold standard imaging modality for the diagnosis of CCFs is a cerebral angiogram; however, less invasive imaging via computed tomography (CT), MRI, or CT/MR angiography are typically performed first.^1^ The goal of CCF treatment is to occlude the fistula while preserving flow through the ICA.^1^,^3^,^4^ Our patient underwent successful transvenous coiling and embolization of his CCF (Image 3), which is the preferred treatment modality for indirect CCFs.^4^ Postoperatively, the patient did well and had no complications.
